# P73 - Speak Up For Asthma (SUFA) Program for schoolchildren in London – a pilot study

**DOI:** 10.1186/2045-7022-4-S1-P128

**Published:** 2014-02-28

**Authors:** Jonny Coppel, Lucy Gibson, Rahul Chodhari

**Affiliations:** 1 University College London Medical School, London, United Kingdom; 2 Royal Free London NHS Foundation Trust, London, United Kingdom

## Introduction

There are three children with asthma in every classroom in English secondary schools. Asthma UK charity predicts that more than 75% of hospital visits are preventable by better recognition of symptoms and early management. Hence, education and empowerment of children is a valuable key in reducing hospital visits.

## Method

A paediatric Consultant trained five senior medical students in conjunction with their Speak Up For Asthma (SUFA) programme. The medical students delivered an interactive presentation to schoolchildren aged 7-14 years. The effectiveness of the programme was measured by comparing results of an asthma awareness test before and after interactive presentation.

## Results

Asthma awareness test had 11 questions with combination of true/false and descriptive statements.

1. The mean score increased from 55% to 93% after the teaching across all ages (p<0.01).

2. Girls pre-course scores were lower than boys but improved to a similar level of >90% after teaching.

3. Nine and ten year olds showed highest improvement in scores in comparison to other age groups.

4. The greatest increases were seen in the questions concerning the use of inhalers, asthma attack triggers and correct asthma attack response.

5. Teachers and school nurses gave positive feedback on asthma update.

## Discussion

Results are encouraging from many viewpoints.

1. SUFA programme represents a cost effective, low investment opportunity to engage curiosity of schoolchildren.

2. Although, in the short-term there was significant improvement in the asthma knowledge of schoolchildren, it would be crucial to establish long-term efficacy of the programme by administering similar test at three and six months.

3. Our results suggest that in its current format the programme confers the most benefit to children aged nine to ten. Alternative ways to engage wider age groups should be studied.

